# Prenatal genomics and autism: detectable susceptibility without validated individual-level prediction

**DOI:** 10.3389/fpsyt.2026.1973963

**Published:** 2026-10-28

**Authors:** Ainaz Shateri

**Affiliations:** Department of Mental Health, Johns Hopkins Bloomberg School of Public Health, Baltimore, MD, United States

**Keywords:** autism spectrum disorder, clinical validity, copy-number variation, genetic counseling, genetic susceptibility, polygenic risk score, prenatal genomics, variants of uncertain significance

## Abstract

Autism spectrum disorder (ASD) is a behaviorally defined, biologically heterogeneous neurodevelopmental condition with high heritability, estimated at 0.83 (95% CI 0.79–0.87) in Swedish population data. Large-scale exome sequencing, copy-number variant analysis, and genome-wide association studies have implicated a growing number of genes and genomic loci in autism liability, and functional genomic work has repeatedly converged on mid-gestational cortical development. In parallel, prenatal genetics has moved from karyotyping to chromosomal microarray analysis, exome and genome sequencing, and screening based on cell-free DNA in maternal plasma. This review asks how far these developments allow genetic susceptibility to autism to be identified before birth, and what prevents such identification from constituting prediction or diagnosis of autism in an individual fetus. It organizes the evidence in four steps — analytic validity (can the variant be detected in fetal DNA)?, variant interpretation (can it be classified under ACMG/AMP and ACMG–ClinGen standards)?, clinical validity (how far does it shift the probability of autism)?, and clinical utility (does knowing it improve outcomes)? — which makes explicit the classification step within the conventional three-stage sequence and locates where translation breaks down. A second distinction, between molecular diagnosis of a genetic condition and diagnosis of autism itself, runs alongside it. Recurrent copy-number variants such as 16p11.2 and 15q11–q13 rearrangements, and pathogenic variants in genes such as CHD8, SCN2A, SHANK3, and PTEN, are technically detectable by currently available prenatal diagnostic genomic platforms when such testing is clinically indicated. Their interpretation is frequently incomplete, their clinical validity for autism specifically is limited by incomplete penetrance, variable expressivity, and pleiotropy, and their clinical utility for neurodevelopmental prediction is unestablished. Common polygenic variation contributes substantially to population-level liability, but individual-level discrimination is poor and ancestry portability is limited. Prenatal diagnostic genomic testing is additionally largely indication-driven rather than a population screen for neurodevelopmental risk. Prenatal sequencing may therefore establish a molecular diagnosis of a condition associated with an increased likelihood of autism; this is not prenatal diagnosis of autism. Progress will require ancestrally diverse cohorts, population-based penetrance estimates, longitudinal prenatal-to-childhood follow-up, and probabilistic multimodal models, alongside counseling standards that resist deterministic interpretation.

## 1 Introduction

Autism spectrum disorder (ASD) is a behaviorally defined neurodevelopmental condition characterized by differences in social communication and by restricted and repetitive patterns of behavior, interests, or activities. It is diagnosed from observed behavior and developmental history rather than from any biological marker, and it encompasses a broad range of cognitive, language, adaptive, and co-occurring medical profiles (1). Family and twin studies demonstrate a large genetic contribution: reanalysis of Swedish population data estimated heritability at 0.83 (95% CI 0.79–0.87), with non-shared environmental influence estimated at 0.17 (95% CI 0.13–0.21) and shared environmental contributions minimal (2). This combination of high heritability with behavioral definition and phenotypic breadth has shaped autism genetics for two decades (3, 4) and continues to structure debate about what genomic information can tell us about an individual (5).

Gene discovery has advanced substantially. A landmark large-scale exome sequencing study analyzed 35,584 samples including 11,986 with ASD and identified 102 ASD-associated genes at a false discovery rate ≤0.1 (6); a two-stage analysis integrating genetic data from 42,607 autism cases identified 60 genes at exome-wide significance, including five newly implicated genes of more moderate effect (7). Genome-wide association meta-analysis of 18,381 individuals with ASD and 27,969 controls identified the first replicable common-variant loci and estimated single-nucleotide polymorphism (SNP) heritability at approximately 11.8% on the liability scale (8). Functional genomic analyses converge on shared developmental contexts, most consistently mid-fetal cortical neurons and the transcriptional and chromatin programs shaping them (9, 10), supporting models in which part of autism biology is established prenatally (11).

Prenatal genetics has advanced in parallel. CMA became a first-line diagnostic test for fetal structural anomalies in many clinical settings after a prospective study in 4,406 pregnancies showed that, among fetuses with a structural anomaly and normal karyotype, CMA identified clinically relevant deletions or duplications in 6.0%, and in 1.7% of pregnancies tested for advanced maternal age or positive screening (12). Prospective cohorts subsequently established incremental diagnostic yield for prenatal ES after non-diagnostic CMA (13, 14), and cfDNA-based testing has extended from common aneuploidies toward selected microdeletions and monogenic conditions (15). Professional bodies now provide explicit guidance on when genome-wide sequencing is appropriate in pregnancy (16).

The convergence of these fields generates an obvious inference that does not follow. Because some ASD-associated variants are detectable in fetal DNA, it is tempting to conclude that autism is becoming detectable before birth. Identifying a variant that raises the population-level probability of a later diagnosis is categorically different from predicting whether a particular fetus will meet diagnostic criteria in childhood — a point emphasized in discussions of clinical translation in autism genetics (17) and in recent critiques of genomic prediction in this condition (18).

Objective, research question, and organizing framework. This review asks: to what extent can current prenatal genomic technologies identify genetic susceptibility to autism before birth, and what biological, predictive, technological, clinical, and ethical limitations prevent these findings from constituting prenatal autism diagnosis? Two conceptual distinctions organize the analysis throughout.

The first concerns how a genomic test is evaluated. Conventional frameworks for evaluating genomic tests distinguish analytic validity, clinical validity, and clinical utility (19). For the purposes of this review we make explicit an intermediate step, variant interpretation, giving four questions rather than three. Analytic validity asks whether a variant can be detected accurately and reproducibly in fetal DNA. Variant interpretation asks whether the detected variant can be classified, that is, whether we know what it does. This step is not a new validity category: it is the classification process already formalized in the ACMG/AMP standards for sequence variants (20) and the ACMG–ClinGen standards for constitutional copy-number variants (21), which we separate here only as an organizing distinction. Clinical validity asks how much, and how reliably, that classified variant changes the probability distribution of the outcome of interest — a graded property reflected in discrimination, calibration, and the uncertainty of estimates rather than a yes/no attribute. Clinical utility asks whether knowing the result improves management or outcomes. Prenatal genomics makes the second stage unusually visible, because sequencing can return accurately detected variants that cannot be classified within the time frame of a pregnancy; separating it from analytic validity is therefore not merely semantic but helps identify an important translational bottleneck in prenatal practice. This four-stage sequence is an organizing device adopted for this review rather than a standardized regulatory taxonomy; its value is that it distinguishes where different barriers to translation arise. The stages are conceptually distinct and frequently conflated, although they are not fully separable in practice: failure at an earlier stage necessarily constrains those that follow. For most ASD-associated variation the answers are, respectively: generally high for established variant classes, frequently incomplete, limited, and unestablished.

The second distinction is between molecular diagnosis and ASD diagnosis. Prenatal sequencing may establish that a fetus carries a pathogenic variant causing a defined genomic or monogenic neurodevelopmental syndrome in which autism occurs at elevated frequency. That is a molecular diagnosis, and it can be a real and clinically actionable finding. It is not a diagnosis of autism, which requires behavioral features that cannot be assessed before birth (1, 17, 18).

The review proceeds as follows. Section 2 describes literature selection. Section 3 examines genomic architecture. Section 4 uses selected high-impact loci to illustrate the gap between analytic detection and clinical validity. Section 5 reviews evidence placing risk genes within prenatal neurodevelopment. Section 6 evaluates prenatal genomic technologies, distinguishing diagnostic testing from screening, and Section 7 works through detection, interpretation, and prediction in turn. Section 8 assesses polygenic scores, Section 9 considers prenatal biological modifiers, and Section 10 synthesizes the argument. Sections 11–14 address counseling, ethics, research priorities, and conclusions, framed by the longstanding aspiration toward precision approaches in neurodevelopmental medicine (22).

A note on terminology. This review uses identity-first language (“autistic people”) alongside diagnostic terminology (“autism spectrum disorder”) where clinical precision requires it. Preferences vary within the autistic community and among clinicians and researchers, and survey evidence from 3,470 UK autism community members found no single way of describing autism that is universally accepted, with some disagreements deeply entrenched (23); the usage here is not intended to imply that either is preferred. Diagnostic terms denote the clinical construct measured in a cited study rather than a characterization of individuals.

## 2 Narrative review approach and literature selection

This narrative review used thematic literature selection and does not claim systematic or exhaustive coverage.

Literature was identified thematically around eight concept groups, combining terms for the condition (autism, autism spectrum disorder, ASD, neurodevelopmental disorder) with terms for: (i) genomic architecture (heritability, exome sequencing, de novo variant, copy-number variant, genome-wide association); (ii) specific loci (CHD8, SCN2A, SHANK3, PTEN, 16p11.2, 15q11-q13); (iii) prenatal neurodevelopment (fetal brain transcriptome, co-expression network, cortical development, organoid); (iv) prenatal genomic technologies (prenatal diagnosis, chromosomal microarray, prenatal exome sequencing, genome sequencing, cell-free DNA, non-invasive prenatal testing); (v) diagnostic yield and systematic reviews of prenatal sequencing; (vi) polygenic scores (polygenic risk score, portability, ancestry, embryo screening); (vii) prenatal biological context (maternal immune activation, maternal infection, cytokine, placenta); and (viii) counseling and ethics (genetic counseling, variant of uncertain significance, reproductive autonomy, neurodiversity).

Within each group, selection prioritized: (i) large primary genomic studies, particularly exome sequencing and association analyses; (ii) prospective prenatal cohort studies reporting diagnostic yield; (iii) systematic reviews and meta-analyses, preferring the most recent authoritative synthesis where one existed; (iv) professional society position statements and clinical practice guidance; and (v) recent conceptual literature on the limits of genomic prediction. Earlier studies were retained where necessary to explain how the field developed — for example the original description of recurrent 16p11.2 rearrangements in autism — rather than for currency of evidence. Where numerical estimates were included, priority was given to estimates reported in the primary publications rather than to figures quoted in secondary sources.

Two limitations follow directly from this approach and should be weighed when reading the synthesis. First, because literature was identified thematically rather than retrieved by a pre-specified, reproducible query with documented screening, selective inclusion cannot be formally excluded, and the balance of evidence presented in any given section reflects editorial judgment. Because selection was thematic, this review does not report databases searched, search strings, search dates, or numbers of records screened, as a systematic review would; accordingly, statements that no study of a given kind was identified refer only to the literature considered here and are not evidence that no such study exists. Second, the coverage of each concept group is uneven by design: prenatal genomic technologies and autism genomic architecture are covered densely, whereas prenatal environmental epidemiology is sampled only to the extent needed to support the argument in Section 9. The conclusions should therefore be read as an interpretive position supported by the cited evidence rather than as a quantitative summary of the field. The literature considered in this review included publications available through August 2026.

## 3 Autism as a highly heritable but genetically heterogeneous condition

### 3.1 Heritability and its interpretation

Autism is among the most heritable neurodevelopmental conditions, with a population-based estimate of 0.83 (2). Estimates of this magnitude are frequently misread as implying determinism at the individual level. Heritability is a population-level variance statistic, conditional on the population and environments sampled; it quantifies how much of the observed variation in liability is attributable to genetic variation in that population, and says nothing directly about the predictability of any individual outcome. High heritability is fully compatible with poor individual-level prediction (3, 17, 18).

### 3.2 De novo and rare variation

Family-based exome sequencing drove the earliest decisive advances. Comparison of probands with unaffected siblings established that de novo likely gene-disrupting variants are significantly enriched in autistic individuals and contribute to a substantial minority of simplex cases (24), while parallel case–control and family analyses converged on functional categories rather than isolated genes — chromatin remodeling, transcriptional regulation, and synaptic function (25). Integration of de novo single-nucleotide variants with de novo CNVs produced a consolidated set of 65 ASD-associated genes and six CNV loci among 71 risk loci (26), findings later synthesized into a framework linking de novo mutation to molecular neuroscience (27).

Scaling has substantially expanded gene discovery without yielding a simple or unified genetic explanation. Satterstrom and colleagues identified 102 ASD-associated genes at a false discovery rate ≤0.1, showing that these are expressed in both excitatory and inhibitory neuronal lineages and are enriched for regulators of gene expression and mediators of neuronal communication (6). Critically for prediction, 49 of the 102 showed higher frequencies of disruptive de novo variants in individuals ascertained for severe neurodevelopmental delay, and 53 in individuals ascertained for ASD (6). Extension to inherited variation, integrating genetic data from 42,607 autism cases, identified 60 genes at exome-wide significance, including five new genes of more moderate effect; loss-of-function variants in the moderate-risk genes were associated with less cognitive impairment than variants in highly penetrant genes such as CHD8, SCN2A, and SHANK3 (7). Two conclusions follow. First, current evidence does not indicate convergence on a small set of genes accounting for most autism liability (6, 7, 28, 29). Second, many of the strongest ASD-associated genes are not autism-specific: they are neurodevelopmental genes whose disruption produces a range of outcomes of which autism is one (6, 7, 17).

Postzygotic (somatic) mosaic variation. Not all de novo variation is present in every cell: postzygotic mutations arise after fertilization and are carried by only a fraction of cells. Reanalysis of exome data from 5,947 ASD families estimated that 7.5% of de novo mutations, detected mostly in blood-derived DNA, were postzygotic, and damaging postzygotic mutations in critical exons of prenatally expressed genes were more frequent in probands than in controls (30). An independent analysis of more than 2,200 Simons Simplex Collection families, using a calling approach designed for low allele fractions, classified 22% of de novo single-nucleotide variants in children as mosaic, found that transmitted parental mosaic mutations accounted for 6.8% of presumed de novo mutations, and estimated that postzygotic mosaic mutations potentially contribute risk in 3–4% of simplex ASD cases (31). The difference between these proportions partly reflects detection: exome sequencing detects mosaic variants with reasonable sensitivity only when they are present in roughly 25–30% or more of cells, and the minimum detectable allele fraction depends on sequencing depth (30, 31). Both studies sampled blood rather than brain. For prenatal genomics, mosaicism therefore adds uncertainty in two directions: the fetal tissue sampled may not reflect the variant fraction in the developing brain — an inference of this review rather than a finding of the cited studies — and an apparently de novo finding may reflect low-level parental mosaicism, with consequences for recurrence-risk counseling (31).

### 3.3 Copy-number variation

Recurrent CNVs occupy an intermediate position between monogenic and polygenic architecture. The reciprocal ~600 kb rearrangement at 16p11.2 was identified in approximately 1% of autism samples in early array-based studies (32) and has become the paradigm for the phenotypic breadth of recurrent genomic disorders (33); CNV loci were formally incorporated alongside sequence-level genes in a unified risk-locus framework (26). Recurrent CNVs are among the ASD-associated variant classes most established in current prenatal diagnostic workflows (12, 34–36), which makes their interpretive properties — incomplete penetrance, variable expressivity, and transmission from minimally affected parents — directly relevant to prenatal practice.

### 3.4 Common variation and polygenic liability

Rare variants of large effect explain only a minority of autism cases and do not account for most population-level genetic liability. The largest ASD genome-wide association meta-analysis identified five genome-wide-significant loci and estimated SNP-based heritability at approximately 11.8% on the liability scale, with individual variants of very small effect (8). Common polygenic variation therefore contributes substantially to population-level ASD liability, while rare variants of larger effect account for a smaller proportion of cases; the precise partition remains unresolved and depends on how liability is defined and estimated. Common and rare variation are not competing models: polygenic transmission disequilibrium analyses demonstrated over-transmission of autism polygenic risk to affected offspring and showed that the two act additively on liability (37). Contemporary syntheses describe an architecture in which a small number of individuals carry a single high-impact variant, a larger number carry moderate-effect variants, and a broad polygenic background contributes across the population (28, 29).

Quantitative contributions of rare and common variation. Published estimates answer different questions and cannot be added to give a single rare-versus-common partition. Total heritability (0.83 in Swedish population data) is the proportion of population variance in liability attributed to all genetic factors (2). SNP heritability — the share of liability variance captured jointly by common genotyped variants — was approximately 11.8% on the liability scale in the largest GWAS meta-analysis (8); it depends on genotyping coverage and the assumed population prevalence and should not be read as the full contribution of common variation. Rare-variant contributions are usually reported instead as the frequency of a variant class among cases compared with controls: in exome data from 63,237 individuals, 3.95% of autistic individuals and 1.39% of unaffected siblings with CNV data carried at least one de novo coding CNV, and damaging de novo coding variants in the 72 genes associated at a false discovery rate ≤0.001 were found in 4.0% of cases and 0.5% of controls (38). Such carrier frequencies are neither the percentage of autism caused by a variant class — part of the case frequency reflects background mutation also seen in controls — nor molecular diagnostic yields, which depend on clinical ascertainment and laboratory classification. With these caveats, the evidence is consistent with common variation accounting for the larger share of population liability, and with rare damaging variants being carried by a minority of autistic individuals while conferring much larger individual effects (37, 38).

### 3.5 Why no single architecture predominates

No single genetic etiology accounts for most autism (6–8, 26, 28, 29). The breadth of this heterogeneity is visible in SFARI Gene, a curated, community-annotated knowledgebase that catalogs genes reported in association with autism and assigns each an evidence score (39). Its 2026 Q2 release lists 1,289 genes — 1,195 scored and 94 not yet categorized; 245 are in Category 1, the highest-confidence tier, and 218 carry the syndromic (S) designation, which can accompany a numeric score (40). Because entries range from high-confidence to suggestive evidence, the catalog is a map of loci at varying evidentiary thresholds rather than a list of confirmed autism-risk genes, and inclusion of a gene does not establish that a variant detected in it prenatally is pathogenic or predictive of autism. Different individuals reach the same behavioral threshold through different combinations of rare and common variation acting on partly overlapping developmental pathways (28, 29, 37). Computational prioritization of additional risk genes (41) can extend the candidate list but cannot resolve the underlying issue: the mapping from genotype to autism phenotype is many-to-one and probabilistic in both directions (17, 28, 29). Any prenatal application inherits this property. A negative genomic result cannot exclude susceptibility, because much liability resides in common variants that can be genotyped but are individually uninformative (8, 37); a positive result identifies a probability shift of uncertain magnitude rather than an outcome (17, 18, 33). **Table 1** summarizes the major classes of ASD-associated variation and their implications for prenatal detection.

**Table 1:** Major classes of genetic variation associated with ASD and their implications for prenatal detection.

Genetic category	Examples	Evidence for ASD association	Analytic detectability (prenatal)	Interpretation and predictive limits	Refs
*De novo* likely gene-disrupting variants	*CHD8*, *SCN2A*, *SHANK3*	Enrichment vs unaffected siblings; 102 genes at false discovery rate ≤0.1	Trio ES or GS, where indicated	Individually rare; 49 of 102 genes more often disrupted in severe developmental delay than ASD; severity not specified by genotype	(6, 24–26)
Inherited rare variants of moderate effect	*NAV3*, *ITSN1*, *SCAF1*, *HNRNPUL2*	*De novo* and inherited rare coding variation jointly identified 60 genes at exome-wide significance in 42,607 autism cases; inherited loss-of-function variation contributed to several moderate-risk genes	ES or GS; parental samples facilitate phasing and inheritance assessment	Generally lower effect size and penetrance than highly penetrant *de novo* neurodevelopmental variants (estimated relative risk ~4 for *NAV3*); often transmitted from unaffected parents; less cognitive impairment than high-penetrance genes	(7)
Recurrent CNVs (genomic disorders)	16p11.2 del/dup; maternal 15q11–q13 dup; 22q13.3 del	Replicated association; included among ASD risk loci	Readily detected by diagnostic CMA	Incomplete penetrance; wide expressivity; unaffected transmitting parents; parent-of-origin effects	(26, 32, 33, 56–60, 66–68)
Rare or private CNVs	Non-recurrent imbalances affecting risk genes	Aggregate burden evidence	CMA or GS	High proportion classified as uncertain; sparse population frequency data	(26, 32, 36)
Syndromic single-gene disorders	*PTEN*; *SHANK3* (Phelan–McDermid)	Cohort studies in ascertained populations	ES, GS, or CMA by variant class	Yields molecular diagnosis, not ASD diagnosis; medical implications beyond neurodevelopment; parental status implications	(51–55)
Common variation/polygenic background	Genome-wide common SNPs	5 genome-wide-significant loci; SNP heritability ≈11.8% on liability scale	Genotypable in principle; no validated prenatal application	Very small effects; overlapping distributions; ancestry bias; no validated threshold identified	(8, 37, 98, 99)
Non-coding and regulatory variation	lncRNAs co-expressed with risk genes	Co-expression and functional genomic evidence	Detectable by GS depending on variant class, genomic region, coverage, and assay performance	Variant interpretation is the barrier: annotation and classification frameworks immature; rarely reportable	(73, 79)

## 4 High-impact ASD-associated genes and genomic regions

Each of the following loci is well supported as an autism-associated locus; each is analytically detectable prenatally; and in each case the molecular finding would leave the eventual behavioral phenotype uncertain. They were selected not because they are the most frequent or most penetrant genetic causes of autism, nor because they are the findings most often encountered prenatally, but because each is well characterized and illustrates a different point at which inference from a detectable fetal variant to an individual outcome weakens: CHD8, a comparatively coherent syndrome without a validated prenatal phenotypic marker; SCN2A, allele-specific consequences that make novel missense variants difficult to interpret; SHANK3/22q13.3, a molecular diagnosis that predicts global developmental impairment more consistently than autism; PTEN, a finding whose clearest implications are oncological and familial; 16p11.2, straightforward detection alongside incomplete penetrance and ascertainment-dependent estimates; and 15q11–q13, parent-of-origin effects that must be resolved before interpretation. **Table 2** summarizes these examples.

**Table 2:** Selected high-impact ASD-associated genes and genomic regions relevant to prenatal genomic detection.

Locus	Nature of ASD association	Penetrance and expressivity	Associated phenotypes	Prenatal detectability	Why detection is not ASD prediction	Refs
*CHD8* (chromatin remodeling)	Among the most reproducibly implicated genes; defines a recognizable subtype	Comparatively coherent phenotype; severity not specified	Macrocephaly, distinctive facies, gastrointestinal disturbance (postnatal features)	ES/GS where an indication exists; within the literature considered in this review, no evidence identified that fetal head circumference is a sensitive or specific prenatal indicator	Molecular diagnosis of a neurodevelopmental syndrome in which ASD is frequent but not invariable	(42–45)
*SCN2A* (NaV1.2 channel)	Established risk gene; allele-specific consequences	Phenotype tends to vary with direction of functional effect, with exceptions	Infantile and later-onset epilepsy syndromes differing in treatment response	ES/GS; detection straightforward	Novel missense variants may remain VUS absent functional or segregation evidence	(46, 47)
*SHANK3*/22q13.3 deletion (postsynaptic scaffold)	Point mutations and terminal deletions; core gene of Phelan–McDermid syndrome	Larger deletions associated with greater severity	Global developmental delay, absent/delayed speech, hypotonia	CMA detects the deletion; ES/GS detects sequence variants	Establishes Phelan–McDermid syndrome; more consistently associated with global developmental impairment than with a specific eventual ASD phenotype	(50, 51)
*PTEN* (PI3K/AKT/mTOR)	Identified in ASD with extreme macrocephaly and in pediatric ASD/developmental delay cohorts	Variable; penetrance for ASD incomplete	Macrocephaly; lifelong tumor predisposition; parental implications	ES/GS where indicated	Establishes PTEN hamartoma tumor syndrome; oncological and familial information alongside uncertain neurodevelopmental outcome	(52–55)
16p11.2 deletion/duplication (~600 kb)	Identified in ~1% of ASD samples in early array-based cohorts (ascertainment-dependent, not a population prevalence estimate); paradigm genomic disorder	Markedly incomplete penetrance; found in individuals with ID without ASD and without apparent phenotype; ASD in 20/85 (24%) of predominantly clinically referred deletion carriers	Developmental delay, epilepsy, behavioral difficulties, reciprocal head size, psychiatric features	Diagnostic CMA; no validated cfDNA screening identified for this locus (63, 64)	Defines a distribution of outcomes including typical development, not a predicted phenotype	(32, 33, 56–61)
15q11–q13 duplication (imprinted)	Consistently ASD-associated when maternally derived	Maternal duplications associated with developmental/autistic phenotypes; paternally derived duplications generally lower penetrance, may occur in unaffected carriers	Developmental delay, epilepsy, hypotonia	CMA, often incidentally; reported in prenatal cohorts	Interpretation requires parental origin; outcomes not uniform	(66–68)

Inclusion in this table indicates an established association with autism and analytic detectability by a prenatal platform. It does not indicate clinical validity for predicting autism in an individual fetus; the final column states why for each locus.

### 4.1 CHD8

CHD8 encodes a chromodomain helicase involved in chromatin remodeling and is among the most reproducibly implicated autism risk genes. Targeted resequencing identified disruptive CHD8 mutations in individuals sharing a recognizable profile — macrocephaly, distinctive facial features, and gastrointestinal disturbance — supporting a CHD8-related subtype defined early in development (42, 43). Mechanistic work explains why the gene is pivotal rather than merely associated: knockdown in human neural progenitors dysregulates neurodevelopmental pathways and other autism-associated genes (44), and genome-wide binding analyses in human neural stem cells and fetal brain show CHD8 targets enriched for additional ASD risk genes and co-expressed during mid-fetal development (45).

Analytically, a de novo loss-of-function CHD8 variant is detectable by prenatal ES or GS where such testing is performed (13, 14). Clinically, it would establish a molecular diagnosis of a neurodevelopmental syndrome in which autism is frequent but not invariable, without specifying severity or cognitive outcome (42, 43). Macrocephaly is a recognized postnatal feature of this phenotype (42, 43); current evidence does not establish fetal head circumference as a sensitive or specific prenatal indicator of CHD8-related disorder, and prenatal ascertainment of CHD8 should therefore not be presented as a realistic consequence of routine ultrasound findings.

### 4.2 SCN2A

SCN2A encodes the voltage-gated sodium channel NaV1.2 and illustrates allele-specific rather than gene-specific consequences. Functional characterization indicates that variants observed in autistic individuals tend to reduce channel function, whereas variants associated with infantile seizures tend to increase it — opposing biophysical effects within one gene producing divergent outcomes (46). This is a general genotype–phenotype tendency with exceptions rather than a deterministic division: loss-of-function variants are also found in individuals whose seizures begin later in childhood, clinical series describe heterogeneous combinations of seizure onset, cognitive outcome, and treatment response, and the functional effect of most novel missense variants has not been measured (46, 47). Clinical series confirm marked genetic and phenotypic heterogeneity in SCN2A-related disorders, with age at seizure onset distinguishing groups that differ in response to sodium channel blockade (47).

The prenatal implication separates analytic validity from interpretability. Detecting many sequence variants in SCN2A is technically feasible with ES or GS; interpreting their phenotypic consequences may be substantially more difficult. A novel SCN2A missense variant may remain classified as a variant of uncertain significance (VUS) when sufficient functional, segregation, population-frequency, or phenotypic evidence is unavailable (46, 47) — a situation the prenatal setting makes more likely, given limited fetal phenotypic information and compressed timelines (48, 49).

### 4.3 SHANK3 and Phelan–McDermid syndrome

SHANK3 encodes a master scaffolding protein of the excitatory postsynaptic density and was among the earliest genes linked to autism through both point mutations and 22q13.3 deletions (50). In Phelan–McDermid syndrome, autism occurs at high frequency alongside global developmental delay, absent or severely delayed speech, and hypotonia, with evidence that larger deletions are associated with greater phenotypic severity (51). A 22q13.3 deletion is readily detected by prenatal CMA (12, 36), and its detection would constitute a molecular diagnosis of Phelan–McDermid syndrome (50, 51). That finding is more consistently associated with global developmental impairment than with a specific eventual ASD phenotype; autism is a frequent but not invariable component, and the genotype–phenotype gradient describes syndrome severity rather than autism specifically (51).

### 4.4 PTEN

PTEN illustrates ascertainment-dependent association and the reach of molecular diagnosis beyond neurodevelopment. Germline PTEN mutations were identified in a subset of individuals with ASD and extreme macrocephaly (52), and subsequent clinical cohorts confirmed PTEN mutations in children with autism, developmental delay, and macrocephaly (53), establishing head circumference as a useful indicator for targeted genetic evaluation (54). A prenatally identified pathogenic PTEN variant would establish the molecular basis of PTEN hamartoma tumor syndrome, conveying information about substantially elevated lifetime risks of breast, thyroid, endometrial, renal, and other cancers (55) and carrying implications for parental genetic evaluation, while the neurodevelopmental phenotype remains variable and penetrance for autism specifically incomplete (52–54).

### 4.5 The 16p11.2 recurrent deletion and duplication

No locus better demonstrates the distance between detection and prediction. The reciprocal 16p11.2 rearrangement was associated with autism in approximately 1% of cases in initial cohorts (32), but phenotypic characterization rapidly complicated the picture. Individuals ascertained for autism carried both de novo and inherited 16p11.2 CNVs, including inheritance from parents who did not meet diagnostic criteria (56). Independent series described the rearrangements in association with global developmental delay, behavioral difficulties, dysmorphism, epilepsy, and reciprocal head-size effects — macrocephaly with deletion, microcephaly with duplication — rather than with autism specifically (57). Most tellingly, 16p11.2 deletions were identified in individuals with intellectual disability who were not autistic, and in individuals without apparent clinical phenotype (58). Mechanistic and phenotypic syntheses now treat 16p11.2 CNVs as broadly acting neurodevelopmental risk factors with incomplete penetrance and highly variable expressivity (33). Deep phenotyping of duplication carriers shows a wide distribution of autistic, attentional, cognitive, and psychiatric features rather than a uniform outcome (59), and clinical reports of inherited microdeletions document transmission through families in which carrier status and clinical presentation are dissociated (60).

Ascertainment and quantitative estimates. The available numbers show why the direction of conditioning and the route of ascertainment matter. Among individuals ascertained for autism, the recurrent ~600 kb BP4–BP5 deletion is found in approximately 0.6% (0.3–1% across studies), compared with an estimated background population prevalence of 0.04–0.05% (61) — an estimate of P(CNV | ASD). The converse quantity, P(ASD | CNV), was examined in the Simons Variation in Individuals Project, which evaluated 85 deletion carriers — predominantly clinically referred after microarray testing ordered for clinical concerns, supplemented by cascade testing of relatives — together with non-carrier family members; experienced clinicians assigned best-estimate DSM-IV-TR diagnoses informed by standardized assessments. ASD was diagnosed in 20 of 85 carriers (24%) — 20 of 78 children and adolescents and none of 7 adults — and in none of the non-carrier family members assessed (61). In a study of the reciprocal duplication, ASD occurred in nearly 20% of child carriers, and the phenotype ranged from asymptomatic presentation to substantial disability (59). These are phenotype-first estimates: most carriers came to attention because they or a relative had clinical difficulties, a design that the authors note is biased against clinically asymptomatic carriers, although they cite population data showing cognitive impairment in unselected carriers as evidence that their cognitive findings are not solely an artifact of ascertainment (61). Genotype-first ascertainment identifies carriers regardless of phenotype. In a random sample of 7,877 adults from the Estonian population biobank, 56 carriers of known genomic disorders — including 4 carriers of the 16p11.2 deletion and 7 of the duplication — were identified without clinical preselection; 31 (55%) had formal diagnoses, and 39 (70%) formal diagnoses or self-reported problems, of kinds previously associated with their CNV (62). These population data concern adult cognitive, educational, and general clinical features and provide no autism penetrance estimate, but they show both that population-identified carriers are not uniformly unaffected and that clinically ascertained percentages cannot be transferred directly to a fetus in whom a CNV is found incidentally. Neither P(CNV | ASD) nor a penetrance figure from a clinically referred cohort is a prenatal individual prediction.

16p11.2 CNVs are readily detectable by diagnostic CMA (12, 36). Although expanded cfDNA platforms may target selected subchromosomal abnormalities, within the literature considered for this review we identified no cfDNA assay validated as an autism-risk screening strategy, and any positive screening finding requires diagnostic confirmation (63–65). Where a 16p11.2 CNV is identified prenatally — a realistic scenario, usually incidental to another indication — the defensible counseling message is a distribution of possible neurodevelopmental outcomes, including typical development, rather than a prediction (33, 56–60).

### 4.6 Maternally derived 15q11–q13 duplication

Duplication of the imprinted 15q11–q13 region is among the more consistently ASD-associated CNVs, with clinical series showing that parent-of-origin governs consequence: maternally derived duplications are associated with developmental and autistic phenotypes, whereas paternally derived duplications have generally shown lower penetrance and may occur in unaffected carriers (66). This locus is unusual in that a mechanistic principle — genomic imprinting — partially explains penetrance. It is also increasingly encountered prenatally, with reports describing genetic analysis and prenatal diagnosis of 15q11–q13 microduplication (67) and prenatal ascertainment in fetal cohorts with reported pregnancy outcomes (68). These reports underline that prenatal detection commonly occurs incidentally, that determining parental origin is essential to interpretation, and that outcomes are not uniform (66–68).

## 5 Autism risk genes during prenatal human brain development

### 5.1 Convergence on mid-fetal cortical development

The strongest biological argument that part of autism originates prenatally comes from mapping risk genes onto human brain developmental transcriptomes. Co-expression networks anchored on high-confidence ASD genes converged on deep-layer cortical projection neurons during mid-fetal development (9), while an independent integrative analysis implicated glutamatergic cortical projection neurons and mid-fetal transcriptional modules, distinguishing the developmental contexts enriched for rare versus common variant signal (10). These analyses depend on reference atlases of the developing human brain: the spatio-temporal transcriptome across brain regions and developmental stages (69) and the laminar transcriptional architecture of the mid-gestational cortex (70). Single-cell analysis spanning prenatal and postnatal human cortical development has since resolved these signals to specific cell types and maturation stages (71). Complementary co-expression approaches identified shared pathways among autism candidate genes in the developing brain (72) and extended analysis to long non-coding RNAs co-expressed with risk genes (73), indicating that convergence is not restricted to protein-coding elements. Genes within the 16p11.2 interval are expressed during human fetal corticogenesis (74), providing a developmental rationale for the CNV’s neurodevelopmental effects.

### 5.2 Mechanistic classes and their developmental timing

The functional categories emerging from sequencing studies map onto identifiable prenatal processes. Chromatin regulation is exemplified by CHD8, whose targets are enriched for other ASD risk genes and whose perturbation in human neural progenitors dysregulates neurodevelopmental programs (44, 45). Synaptic scaffolding genes such as SHANK3 act later, at synapse formation and maturation (50). Models of early cell-cycle and proliferation dysregulation propose that altered neural progenitor behavior in the first and second trimesters contributes to subsequent phenotypic severity (11). Mouse cortical organoid models of monogenic ASD allow prenatal-equivalent developmental processes to be manipulated and observed directly (75); translation from such model systems to human clinical outcomes remains indirect, and model-system evidence should not be equated with human clinical evidence.

### 5.3 Prenatal correlates and their limits

If ASD-associated variation acts prenatally, prenatal phenotypic correlates might be expected. Evidence is emerging but limited: an association has been reported between rare ASD-linked genetic variants and ultrasonographically detected fetal anomalies among children later diagnosed with autism (76). These are group-level associations in ascertained samples and do not establish that fetal imaging can identify pregnancies in which the child will later meet diagnostic criteria for autism. Critical commentary argues more broadly that framing autism as a condition established in utero, while biologically defensible, does not license genomic prediction and may obscure the extent of post-genomic developmental variability (18).

The correct inference is narrow but real: multiple independent lines of evidence place a substantial component of autism biology within prenatal cortical development (9–11, 45, 69–71). That is a statement about when the biology operates, not about when the phenotype can be identified.

## 6 Current prenatal genomic technologies

Prenatal diagnostic genomic testing is principally indication-driven, whereas cfDNA is used as a screening modality. Diagnostic testing is offered for fetal structural anomalies, abnormal screening results, family history of a known genetic condition, or parental carrier status (16, 36, 77, 78). A distinction that runs through this section, and that is not always made explicit in the literature, is between diagnostic testing — CMA, ES, and GS performed on fetal material obtained by chorionic villus sampling or amniocentesis (12, 36, 79) — and screening, principally cfDNA analysis of maternal plasma, which estimates risk and requires diagnostic confirmation (15, 63–65). **Table 3** compares the platforms on this basis.

**Table 3:** Current and emerging prenatal genomic technologies.

Technology	Diagnostic or screening	What is detected	Current prenatal role	Relevance to ASD-associated variation	Major limitations	Refs
Chromosomal microarray analysis (CMA)	**Diagnostic** (chorionic villus sampling or amniocentesis)	Genome-wide copy-number gains and losses below karyotype resolution	First-line test for fetal structural anomalies	Detects recurrent ASD-associated CNVs relevant to current prenatal practice	No sequence variants or balanced rearrangements; appreciable rate of uncertain copy-number findings; invasive sampling	(12, 34–36)
Exome sequencing (ES)	**Diagnostic** (chorionic villus sampling or amniocentesis)	Coding sequence variants; trio design enables *de novo* detection	Second-line after non-diagnostic CMA in anomalous fetuses	Detects pathogenic variants in high-confidence ASD genes	Yield strongly phenotype-dependent (8.5–10.3% in prospective fetal-anomaly cohorts; 27% pooled incremental yield over CMA in the 2026 systematic review); substantial VUS burden; not indicated without fetal or familial indication	(13, 14, 16, 77, 78, 80–86)
Genome sequencing (GS)	**Diagnostic** (chorionic villus sampling or amniocentesis)	Coding and non-coding variants, structural variants, copy number in one assay	Emerging; selected centers	Broadest technical coverage of ASD-associated variant classes; broader coverage does not necessarily mean higher reportable yield	Greatest interpretive burden; non-coding classification immature; incremental yield over ES modest in some comparisons	(79, 87, 88)
Cell-free DNA (non-invasive prenatal testing)	**Screening** (maternal plasma)	Placenta-derived cfDNA reflecting the fetal/placental genome; aneuploidies, selected microdeletions, selected monogenic conditions	Widely implemented screening test for common aneuploidies; positive results require diagnostic confirmation; routine general-population screening for microdeletions or genome-wide CNVs not recommended (96, 97)	Selected ASD-relevant microdeletions (e.g. 22q11.2) targeted; broader CNV panel performance variable	Not diagnostic; positive predictive value limited in low-prevalence conditions; no assay identified that is validated for population screening across ASD-associated CNVs or as an autism-risk strategy; current guidance directs patients seeking fetal copy-number information to diagnostic testing rather than cfDNA microdeletion screening	(15, 63–65, 89–97)
Polygenic scoring of fetal DNA	Neither (research only)	Aggregate common-variant liability	Not in clinical prenatal use	Theoretically applicable to autism liability	No demonstrated individual-level validity or prenatal utility; ancestry bias; no validated action threshold	(8, 37, 98, 99, 103, 105)

Bold text indicates the classification of each technology as diagnostic or screening.

### 6.1 Chromosomal microarray analysis (diagnostic)

CMA detects sub-microscopic copy-number gains and losses genome-wide at resolution far exceeding karyotyping. The pivotal prospective comparison in 4,406 pregnancies established the yields cited above (12). Systematic appraisal supported the incremental clinical utility of microarray over normal karyotype in prenatal cytogenetics (34), and a prospective cohort with accompanying meta-analysis reached concordant conclusions in anomalous fetuses (35). CMA is now a first-line prenatal diagnostic test in many settings (36).

For autism-relevant purposes, CMA remains a particularly relevant currently deployed platform because it detects the recurrent CNVs — 16p11.2, 15q11–q13, 22q13.3 — which are among the ASD-associated variant classes most established in current prenatal diagnostic practice. Its limitations matter equally: it does not detect sequence-level variants, balanced rearrangements, or most single-gene causes, and it generates copy-number findings of uncertain significance at appreciable frequency (12, 34–36).

### 6.2 Exome sequencing (diagnostic)

Two prospective cohorts established the incremental value of ES after non-diagnostic CMA in anomalous fetuses. The PAGE study identified a diagnostic genetic variant in 52 of 610 fetuses (8.5%, 95% CI 6.4–11.0), with a further 3.9% carrying a VUS of potential clinical usefulness (13); a parallel trio-based cohort of 234 fetus–parent trios reported a diagnostic yield of 10.3%, rising with the number of anomalies present (14). Meta-analysis of 72 reports from 66 studies representing 4,350 fetuses estimated a pooled incremental yield of ES over karyotype/CMA of 31% (95% CI 26–36%), with yield significantly higher in cases pre-selected for likelihood of monogenic etiology than in unselected cases (42% vs 15%) and ranging from 53% for isolated skeletal abnormalities to 2% for isolated increased nuchal translucency (80). Consistent with this, incremental yield reaches 40% in fetuses with recurrent structural anomalies across pregnancies (81) and is elevated in multisystem malformations (82), with organ-system-specific estimates reported for fetal brain anomalies detected by ultrasound and MRI (83, 84) and for skeletal abnormalities (85). An updated 2026 systematic review, extending the earlier search to January 2025, estimated an overall incremental diagnostic yield of sequencing over non-diagnostic CMA of 27%, rising to 40% in selected cohorts and falling to 20% and 17% in unselected and exploratory cohorts respectively (86). Diagnostic yield therefore varies substantially with case selection and phenotype (80–86); and none of these figures describes neurodevelopmental prediction, since all measure molecular diagnosis of the presenting structural phenotype.

Clinical guidance has evolved accordingly, emphasizing case selection, trio testing, rapid turnaround, and pre-test counseling (77), with national working groups issuing opinion statements on indications and reporting (78) and the International Society for Prenatal Diagnosis setting out when genome-wide sequencing is justified in pregnancy while cautioning against untargeted use (16). Prenatal ES and GS are currently used primarily in pregnancies with fetal structural anomalies, particularly after non-diagnostic karyotyping and CMA, or in selected familial contexts; indications vary by jurisdiction, institution, and research setting, and routine sequencing of unselected pregnancies for neurodevelopmental risk prediction is not recommended by current guidance (16, 77, 78).

### 6.3 Genome sequencing (diagnostic)

GS extends coverage to non-coding regions, structural variation, and copy number within a single assay (79, 87, 88). Direct comparison in prenatal cases indicates that GS can identify variants not detected by exome-based approaches, though the incremental gain must be weighed against interpretive burden (79). Prospective implementation in prenatally detected congenital malformations demonstrates clinical feasibility (87), and cohort analyses report yields broadly comparable to or modestly exceeding ES (88). For ASD-associated variation GS offers in principle the broadest technical coverage of any prenatal platform — detecting CNVs, sequence variants, and structural rearrangements simultaneously — and correspondingly has substantial potential to generate findings that are analytically valid but not clinically interpretable. Broader technical coverage does not necessarily mean a higher clinically reportable yield, because sensitivity, validation, and interpretive frameworks differ across variant classes and laboratories (79, 88).

### 6.4 Cell-free DNA and non-invasive prenatal testing (screening)

Analysis of circulating cell-free DNA in maternal plasma has transformed prenatal screening for common aneuploidies and is extending to sub-chromosomal and monogenic targets (15). International implementation has been rapid and uneven, with persisting variation in counseling standards and regulatory oversight (89). Technical reviews describe the trajectory from aneuploidy screening toward single-gene conditions (90), the methodological requirements for non-invasive prenatal diagnosis of monogenic disorders (91), and plasma DNA and RNA sequencing approaches (92).

Extension to microdeletions and microduplications is where cfDNA intersects most directly with autism-associated variation, and where its limits are clearest. Systematic reviews of cfDNA screening for microdeletions and microduplications report widely varying performance and caution about positive predictive value in low-prevalence conditions (63, 64). The best-characterized target is the 22q11.2 deletion: prospective first-trimester screening (93), targeted microarray-based cfDNA approaches (94), and large clinical cohorts (95) have demonstrated feasibility, and a large prospective multicenter study reported high specificity with substantially lower positive predictive value, requiring diagnostic confirmation of all screen-positive results (65). Two conclusions follow. First, cfDNA is a screening modality: it does not diagnose, and performance depends heavily on target prevalence (63–65). Second, within the literature considered for this review we identified no cfDNA assay validated for population screening across the range of ASD-associated CNVs, and none validated as an autism-risk screening strategy.

Professional guidance has moved in the same direction. Society for Maternal-Fetal Medicine Consult Series #74, endorsed by the American College of Obstetricians and Gynecologists (ACOG), and ACOG’s January 2026 Practice Advisory recommend against routine general-population cfDNA screening for any microdeletion condition and against routine cfDNA testing for large genome-wide copy-number deletions or duplications; pregnant patients who want information about fetal copy-number variants are to be offered diagnostic testing rather than cfDNA microdeletion screening (96, 97). The distinction this draws — between what cfDNA can technically interrogate and what it is currently indicated to interrogate — matters for the argument developed here. Even where an ASD-associated CNV lies within the technical reach of an expanded cfDNA panel, current guidance directs the patient who wants copy-number information toward chorionic villus sampling or amniocentesis with CMA. Non-invasive ascertainment of ASD-associated copy-number variation is therefore constrained not only by assay performance and positive predictive value, but by the structure of recommended prenatal care.

## 7 Prenatal detectability of ASD-associated genetic variation

### 7.1 Analytic validity: what can be detected

Three categories of ASD-associated finding fall within the reach of current prenatal diagnostic testing: recurrent CNVs such as 16p11.2, maternally derived 15q11–q13 and 22q13.3, accessible to CMA (12, 34–36); pathogenic sequence variants in high-confidence genes such as CHD8, SCN2A, and SHANK3, accessible to ES or GS (13, 14, 79); and syndromic, pleiotropic variants such as those in PTEN, in which autism is one component of a wider medical phenotype (52, 53). The constraint is not platform capability but ascertainment: because testing is conditional on an existing structural or familial indication (16, 77, 78), these findings arise opportunistically rather than through any systematic search for autism susceptibility.

What cannot currently be translated into validated prenatal prediction is equally important: the polygenic background contributing substantially to population-level liability, which is genotypable in principle but has no validated prenatal application (8, 37, 98, 99); the developmental, environmental, and stochastic contributions that shape whether liability is expressed (100–102); and the behavioral phenotype itself (17, 18).

### 7.2 Variant interpretation: detection is not classification

Analytic detection is not the same as classification. A nucleotide change may be detected with high accuracy by ES or GS and still not be classifiable as pathogenic, benign, or ASD-associated. Classification follows established standards: the five-tier ACMG/AMP framework for sequence variants (20) and the points-based ACMG–ClinGen framework for constitutional CNVs, which applies to prenatal as well as postnatal testing (21). These standards weigh several kinds of evidence, and only some depend on the fetal phenotype. Population frequency, predicted and experimentally measured functional effect, and gene or region dosage sensitivity can be assessed largely independently of the individual tested, whereas criteria that credit a phenotype as highly specific for the gene or region, and de novo or case-level evidence weighted by phenotypic consistency, depend on a phenotype that before birth is limited to imaging findings and cannot include behavior (20, 21). Classification and clinical validity are also distinct: a CNV can be classified as pathogenic when its penetrance and expressivity are well documented “even if reduced and/or variable” (21), so a pathogenic classification does not by itself indicate how likely autism is. This gap is widest for novel missense variants in genes with allele-specific consequences such as SCN2A, where the direction of functional effect determines phenotype and is usually unknown for a novel allele (46); for rare or private CNVs, where population frequency data are sparse; and for non-coding and regulatory variation, where sequence-level detection by GS substantially outpaces interpretive frameworks (73, 79). Prenatal sequencing generates variants of uncertain significance (VUS) at rates that materially affect clinical utility, and whether and how to report them prenatally remains actively debated (48, 49). A VUS in a plausible neurodevelopmental gene, discovered mid-pregnancy, is among the most difficult results in clinical genomics, and may not be resolvable within the time frame of a pregnancy (48, 49). Any account of prenatal capability that stops at detectability therefore overstates what is available to families.

### 7.3 Clinical validity: classification is not prediction

For each detectable category the obstacles are structural rather than technical.

Incomplete penetrance. A substantial proportion of carriers of recurrent ASD-associated CNVs do not meet criteria for autism. This is documented directly for 16p11.2, where deletions have been identified in individuals with intellectual disability but not autism and in individuals without apparent phenotype (58), and where carriers are frequently ascertained through unaffected transmitting parents (56, 60).

Variable expressivity. Where a phenotype is present, its nature and severity vary widely across carriers of the same variant, spanning developmental, epileptic, cognitive, psychiatric, and autistic domains in varying combinations (33, 57, 59).

Pleiotropy and diagnostic non-specificity. Of the 102 genes identified in the large-scale exome study by Satterstrom et al., 49 were more frequently disrupted in individuals ascertained for severe neurodevelopmental delay than for autism (6). A prenatal finding therefore typically indicates elevated neurodevelopmental risk broadly construed.

Parent-of-origin and inheritance effects. For imprinted regions such as 15q11–q13, interpretation depends on determining parental origin (66), and prenatal series demonstrate the practical difficulty of doing so under time pressure (67, 68).

A prenatally identified ASD-associated variant therefore shifts a probability distribution by an amount that is often poorly quantified and rarely quantifiable for the individual fetus (17, 18, 33, 56, 58, 59). Clinical validity in this sense is graded rather than binary: the relevant questions are how far a finding moves the distribution of outcomes from the population baseline, how well it separates children who will and will not meet diagnostic criteria (discrimination), whether stated probabilities match observed frequencies (calibration), and how wide the uncertainty is for the population to which the fetus belongs. For most prenatal ASD-associated findings, the first can be described only approximately and largely from clinically ascertained samples (Section 4.5), and the remaining three have not been established. Such a finding can nonetheless be meaningful clinical information and may constitute a molecular diagnosis.

## 8 Polygenic risk scores and prenatal risk prediction

### 8.1 What a polygenic score is, and what autism association studies support

A polygenic risk score (PRS) aggregates the estimated effects of many common variants into a weighted sum representing an individual’s relative genetic loading for a trait. For autism, PRS construction rests on genome-wide association studies identifying common-variant associations of very small individual effect and SNP heritability of approximately 11.8% on the liability scale (8), and on the demonstration that this polygenic component is over-transmitted to affected offspring and acts additively with rare variation (37). Methodological reviews describe the transition of PRS from research instrument toward possible clinical application (103, 104) and the criteria by which such a transition should be judged (105).

### 8.2 Clinical validity for autism

Current autism PRS do not meet these criteria for individual-level prenatal clinical application. Meta-analysis of phenotypic correlates of the autism polygenic score indicates associations that are statistically detectable but small in magnitude, distributed across cognitive, educational, and behavioral domains rather than specific to autism diagnosis (98). Population studies further show that associations between polygenic scores and autistic traits vary across geographic and social contexts within a single country, indicating that measured associations are not purely biological and do not transfer straightforwardly across settings (106). Small group-level effects translate into wide, heavily overlapping individual distributions: PRS distributions overlap substantially between autistic and non-autistic populations, limiting individual-level discrimination (98, 103–105).

Quantitative estimates from a large population-based sample show the scale of these effects. In a Danish case–control study of 11,339 autistic individuals and 20,175 controls, restricted to individuals of European ancestry with Danish-born parents, the adjusted odds ratio for autism in the top versus the bottom decile of autism PRS was 2.35 (95% CI 2.11–2.63), adjusting for sex, birth year, parental ages, having a sibling, and five ancestry principal components; it was 2.14 (95% CI 1.91–2.40) after further adjustment for psychiatric family history, whereas a family history of autism carried an adjusted odds ratio of 6.73 (95% CI 5.89–7.68) (107). Adding the PRS to a model containing these covariates and psychiatric family history increased variance explained on the liability scale from 0.14 to 0.15 (Nagelkerke R² from 0.22 to 0.23). An odds ratio comparing extreme deciles is neither an individual’s absolute probability of autism nor a measure of prenatal predictive performance. That study did not report discrimination (AUC) or calibration and did not analyze other ancestry groups; within the evidence considered in this review, autism-specific discrimination, calibration, and cross-ancestry performance have not been established for prenatal use, and the incremental value beyond family history reported here is small.

### 8.3 Ancestry bias and portability

PRS performance degrades when scores derived in one ancestral population are applied to another, and the great majority of underlying association-study data derive from European-ancestry participants (99). Any prenatal application would therefore produce systematically less accurate estimates for pregnancies in under-represented populations, converting a research inequity into a clinical one (108, 109).

### 8.4 Clinical utility

Clinical validity concerns whether a score reliably associates with the outcome; clinical utility concerns whether using it improves decisions or outcomes. Autism PRS currently has limited validity at the individual level and no demonstrated prenatal utility (103, 105, 108). In the literature considered for this review, we identified no validated threshold at which a prenatal autism PRS would justify a clinical action, and no prospective study evaluating prenatal clinical use of autism PRS.

### 8.5 Polygenic embryo screening — related but distinct

Polygenic embryo screening applies polygenic scores to preimplantation embryos during in-vitro fertilization. This differs conceptually and practically from prenatal testing in an established pregnancy: it involves selection among embryos rather than decisions about a continuing pregnancy, and its statistical properties differ because selection operates within a sibship. Modeling indicates that expected gains from selecting embryos on polygenic scores are modest and accompanied by considerable uncertainty (110). The practice is nonetheless offered commercially, and survey data from reproductive medicine physicians reveal heterogeneous and often under-specified decision criteria (111). Ethical analyses document the range of arguments advanced for and against clinical polygenic scoring (112) and the attitudes of stakeholders encountering it (113), alongside broader concerns about interpretation and misuse of polygenic risk information for multifactorial conditions (109). The relevance here is indirect: this raises concern that the commercial availability of polygenic selection may generate pressure toward analogous prenatal claims that the underlying science does not support.

## 9 Prenatal biological context and potential gene–environment interactions

Direct evidence for variant-specific gene–environment interaction in human prenatal autism risk remains limited (100, 114–117); the evidence reviewed here is included only to establish that genomic liability develops within a biologically variable prenatal environment, which further weakens deterministic genomic prediction.

Maternal immune activation — activation of the maternal immune system by infection or other inflammatory stimuli during pregnancy — is the most extensively studied example. Preclinical and translational studies describe effects on offspring brain development and behavior, with proposed pathways involving cytokine signaling, IL-6-mediated placental endocrine changes, placental inflammation, and immune regulation at the maternal–fetal interface (114–116, 118–121).

Human evidence is observational and more qualified. Meta-analyses and cohort studies report modest associations of maternal infection, fever, and hospitalization with infection with autism in offspring, heterogeneous by exposure type, timing, and severity (122–126); differences in mid-gestational maternal cytokine and chemokine profiles have been reported (127, 128), some inflammatory associations extend to neurodevelopmental outcomes generally rather than autism specifically (129), and maternal anti-fetal brain autoantibodies have been associated with maternal metabolic conditions (130). A critical appraisal concludes that, while associations are robust and mechanisms plausible, the human evidence does not establish causation for autism (100).

Transcriptomic and pathway analyses report that maternal immune activation perturbs fetal brain gene expression in patterns overlapping autism-implicated genes and the developmental windows identified in Section 5 (101, 131, 132), and broader syntheses reach a similar structural conclusion (102, 117). These are convergence findings, not demonstrations of statistical interaction between specific ASD-associated variants and specific prenatal exposures in humans.

For prediction, the implication is limited but direct: developmental and environmental factors may contribute additional, currently unmeasured variation in the phenotypic expression of genetic liability (100–102, 117). Because such factors are generally unmeasured when prenatal genomic testing occurs and their causal status remains uncertain (100), they cannot presently be incorporated into an individual prenatal risk estimate — an inference of this review rather than an established finding — and they constitute an additional source of uncertainty around genotype-based prediction that improved measurement, as set out in Section 13, might in principle reduce.

## 10 Discussion: why prenatal genomic detection is not prenatal autism diagnosis

Read through the four-stage sequence set out in the Introduction, the position is as follows. **Table 4** maps the corresponding barriers and research priorities onto the four stages and an overarching conceptual distinction, and **Figure 1** summarizes the inferential chain from variant detection to possible clinical action.

**Table 4:** Barriers to prenatal genomic prediction of ASD, mapped to the four-stage evaluation framework, with research priorities.

Stage	Barrier	Why it limits prenatal prediction	Research priority	Refs
Analytic validity — can the variant be detected accurately?	Assay- and specimen-dependent performance	Detection accuracy, coverage, mosaicism, and structurally complex rearrangements vary by platform, laboratory, and sample type	Standardized prenatal assay validation and reporting of analytic performance	(12, 36, 79)
Analytic validity	Incomplete genomic coverage	Repeat expansions, low-coverage regions, and balanced rearrangements are missed by some platforms	Broader adoption of GS where clinically justified	(79, 87, 88)
Variant interpretation — can the variant be classified?	Variant classification lags detection	Some accurately detected variants cannot be classified under ACMG/AMP or ACMG–ClinGen criteria within a pregnancy; phenotype-dependent evidence is limited before birth	High-throughput functional assays; agreed prenatal VUS reporting policy	(20, 21, 46, 48, 49)
Variant interpretation	Non-coding and regulatory variation	Detected by GS depending on variant class, region, and coverage, but annotation and classification frameworks are immature	Regulatory variant annotation in fetal brain contexts	(73, 79)
Variant interpretation	Rare and private CNVs	Sparse population frequency data prevent confident classification	Population reference CNV catalogs across ancestries	(26, 32, 36)
Clinical validity — how much does it shift the probability of ASD?	Extreme genetic heterogeneity	Hundreds of genes implicated at varying evidentiary thresholds; no single locus accounts for a large proportion of autism cases	Continued large-scale, ancestrally diverse discovery	(6, 7, 26, 38)
Clinical validity	Polygenic architecture	Substantial liability sits in common variants of negligible individual effect	Joint rare–common modeling in the same cohorts	(8, 37)
Clinical validity	Incomplete penetrance	Many carriers never meet diagnostic criteria	Population-scale follow-up of non-ascertained carriers	(56, 58, 60)
Clinical validity	Variable expressivity	The same variant produces different phenotypes	Standardized lifespan phenotyping of carrier cohorts	(33, 57, 59)
Clinical validity	Pleiotropy and non-specificity	Top ASD genes often index general developmental delay rather than autism	Cross-disorder analyses quantifying outcome-specific risk per variant	(6)
Clinical validity	Developmental and prenatal biological modifiers	Prenatal biological factors may contribute to variation in phenotypic expression; human causal and interaction evidence remains limited	Longitudinal prenatal-to-childhood cohorts with paired sampling	(100–102)
Clinical validity	Ancestry bias	Reference data, classification, and PRS skewed toward European ancestry	Cohort diversification; ancestry-robust score construction	(99, 108)
Clinical utility — does knowing improve outcomes?	No prenatal clinical utility identified within the literature considered in this review	Effects of returning ASD-susceptibility information on child and family outcomes have not, within that literature, been prospectively established; this does not bear on the separate utility of a molecular diagnosis (Section 10.4)	Prospective evaluation of disclosure and counseling models	(48, 133)
Clinical utility	No clinically validated ASD-specific prenatal biomarker identified	Within the literature considered in this review, no imaging, biochemical, or cfDNA marker is validated for individual-level prediction	Biomarker discovery in prospective cohorts with pre-specified outcomes	(17, 18, 63–65, 76)
Conceptual	Molecular diagnosis ≠ ASD diagnosis	ASD is defined by postnatal behavioral criteria that cannot be assessed prenatally	Counseling frameworks that formalize the distinction	(1, 17, 18)

The “Barrier” and “Why it limits prenatal prediction” columns are supported by the cited references. The “Research priority” column represents the synthesis and recommendations of the present review rather than findings reported in those references.

**Figure 1:**
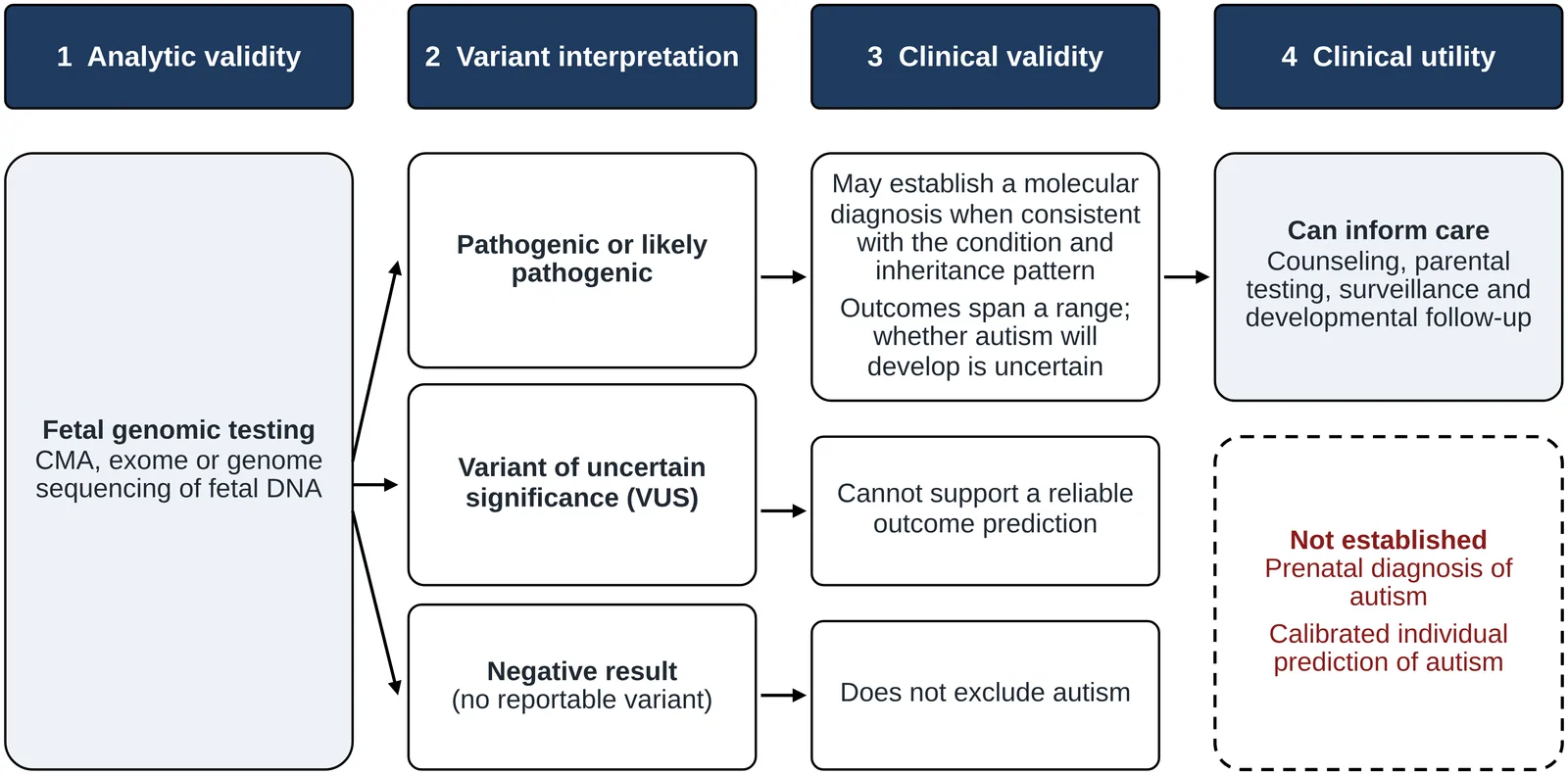
From fetal genomic testing to possible clinical action, following the four steps used in this review. Fetal DNA is tested by chromosomal microarray analysis (CMA), exome sequencing, or genome sequencing (analytic validity), and any variant is classified under ACMG/AMP and ACMG–ClinGen standards (variant interpretation) (20, 21). Cell-free DNA (cfDNA) is a screening test; positive results require diagnostic confirmation. A pathogenic or likely pathogenic finding may establish a molecular diagnosis when consistent with the condition and inheritance pattern; the outcomes that follow span a range, and whether autism will develop remains uncertain (clinical validity). A variant of uncertain significance cannot support a reliable outcome prediction, and a negative result does not exclude autism. A molecular diagnosis can inform care, whereas prenatal diagnosis of autism and calibrated individual prediction of autism are not established (clinical utility).

### 10.1 Analytic validity

Analytic validity is generally high for established variant classes interrogated by validated prenatal workflows, and where it fails — mosaicism, structurally complex rearrangements, repeat expansions, poorly covered regions — the limitations are assay- and technology-dependent rather than consequences of autism’s genetic architecture (12, 36, 79, 87, 88). This stage is therefore the least of the four barriers, and the one most likely to yield to engineering effort.

### 10.2 Variant interpretation

A clinically important subset of accurately detected variants cannot be confidently classified under ACMG/AMP or ACMG–ClinGen criteria within the time frame of a pregnancy (20, 21, 48, 49). The pattern recurs across variant classes for different reasons — absent allele-level functional data, immature annotation of regulatory sequence, insufficient population frequency data for private CNVs — but the consequence is the same: for uncertain findings, interpretation rather than detection may become the principal bottleneck.

### 10.3 Clinical validity

Even for confidently classified variants, clinical validity for autism specifically — how much, and how reliably, a finding shifts the distribution of outcomes — is limited, and six properties of autism’s architecture constrain it in ways that compound rather than merely accumulate. Heterogeneity means that hundreds of genes have been implicated at varying evidentiary thresholds — for example, 72 genes at a false discovery rate ≤0.001 and 185 at ≤0.05 in one joint rare-variant analysis (38) — with no single locus accounting for a large proportion of autism cases (6, 7, 26). Polygenicity means a substantial share of population liability sits in common variants of individually negligible effect, genotypable but individually uninformative, and not currently interpretable as a discrete prenatal finding or quantifiable at the individual level (8, 37, 98). Incomplete penetrance means that many carriers do not develop autism (56, 58, 60). Variable expressivity means those who do vary widely in presentation (33, 57, 59). Pleiotropy means the strongest signals often index general neurodevelopmental risk rather than autism: 49 of the 102 genes identified by Satterstrom et al. were more frequently disrupted in individuals ascertained for severe developmental delay (6). And developmental and environmental factors may contribute additional unmeasured variation in phenotypic expression (100–102). Ancestry bias in reference data, variant classification, and polygenic scores degrades accuracy unevenly across populations (99, 108).

### 10.4 Clinical utility

Within the literature considered in this narrative review, we identified no study demonstrating that returning prenatal ASD-risk information improves outcomes for children or families. No ASD-specific prenatal biomarker has been clinically validated for individual-level prediction; reported associations between ASD-linked variants and fetal ultrasound anomalies are group-level findings in ascertained cohorts (76). This conclusion concerns the utility of prenatal autism prediction. It should be distinguished from the utility of a molecular diagnosis, which does not depend on predicting autism: a pathogenic finding may inform parental testing and recurrence-risk counseling, surveillance for associated medical features such as tumor risk in PTEN hamartoma tumor syndrome, neonatal management, and planned developmental follow-up (16, 55).

### 10.5 Competing schools of thought and unresolved controversies

The evidence reviewed here is interpreted differently across research communities, and these competing perspectives warrant explicit consideration.

High-effect genetic informativeness versus probabilistic phenotypic prediction. One position emphasizes that autism is among the most heritable neurodevelopmental conditions (2) and that specific high-impact variants confer large relative risks (6, 7), and infers that genotype is therefore substantially informative about outcome. A second position emphasizes that heritability is a population variance statistic, that most implicated genes are not autism-specific, and that much liability is distributed across many variants of small effect (8, 28, 29, 37); on this reading, confidence in genotype-based prediction of an individual phenotype should be correspondingly lower. The disagreement is one of degree rather than of kind — neither position holds that autism is genetically determined — and the penetrance data reviewed in Section 4 constrain how much confidence the first position can support (33, 56, 58, 60).

Enthusiasm versus skepticism about genomic prediction. Methodological reviews describe polygenic scores as moving from research instruments toward clinical application (103–105), while empirical evaluations of autism polygenic scores report small effects, heavy distributional overlap, and context-dependent associations (98, 106). These positions are not straightforwardly reconcilable, and the disagreement is partly about which endpoint is being predicted: population stratification and individual clinical prediction are different tasks with different evidentiary requirements.

Scientific possibility versus ethical acceptability. Even where a technology performs as claimed, its appropriateness is contested. Analyses of polygenic embryo screening (110), of the ethics of probabilistic autism genetics (134), and of the disability-rights critique of prenatal testing (135) reach different conclusions about whether such applications should be offered at all, independent of their accuracy.

### 10.6 The categorical distinction

Underlying all four stages is a distinction of kind rather than degree. Autism cannot currently be diagnosed in a fetus because its diagnostic criteria depend on postnatal developmental and behavioral features that cannot be assessed before birth (17, 18). A prenatal finding may therefore establish a molecular diagnosis — Phelan–McDermid syndrome, PTEN hamartoma tumor syndrome, a 16p11.2 or 15q11–q13 genomic disorder — while leaving the question of autism open. Conflating the two is the central error this review is written against.

## 11 Clinical and genetic counseling implications

Postnatal practice offers a partial model. Earlier American College of Medical Genetics and Genomics guidance established a tiered etiological evaluation for autism with CMA and fragile X testing among first-line investigations (136); subsequent evidence and contemporary neurodevelopmental genetics practice have increasingly expanded the role of ES and GS (137, 138). Prenatal counseling differs in three consequential respects: it occurs under time pressure, it concerns a phenotype that cannot yet be observed, and its results may inform decisions about continuing a pregnancy (48, 139, 140).

Naming the finding accurately. Where a molecular diagnosis is established, it should be communicated as such — a named genetic condition with a described phenotypic range — rather than as an autism finding. Existing prenatal counseling guidance emphasizes accurate framing of what a genomic result does and does not establish (16, 48, 78). This framing is more accurate, more actionable, and less prone to deterministic misreading, and it directs attention to what a molecular diagnosis can support — parental testing, recurrence-risk counseling, surveillance, and developmental follow-up — none of which depends on predicting autism.

Risk communication. Results should be communicated probabilistically, with relative and absolute risk distinguished where data permit and clearly labeled as uncertain where they do not. For recurrent CNVs, honest communication describes a distribution of possible outcomes including typical development (33, 59).

Uncertainty as the primary finding. For many prenatal results the dominant content is uncertainty itself. Analyses of prenatal ES identify the management of uncertainty as the central counseling challenge (48). The clinical utility of reporting prenatal VUS remains context-dependent and debated, with some national position statements opposing the practice; recent evidence from a laboratory operating a multidisciplinary review process, however, indicates that carefully selected VUS fitting the fetal phenotype can provide clinically useful information, with subsequent follow-up resolving the uncertainty in over half of cases (49).

Secondary findings and consent. Prenatal sequencing can reveal findings unrelated to the indication, including adult-onset conditions and parental carrier or affected status — as with PTEN — raising distinct disclosure questions (139). Consent should establish in advance what categories of result will be returned, the likelihood of uncertain results, the limits of neurodevelopmental prediction, and the possibility of findings about parents; position statements provide a framework (16, 78).

Psychological consequences. A systematic review of the psychosocial impacts of autism-related genetic testing indicates that effects on families are variable and depend heavily on how results are framed and supported (133). These findings support structured counseling rather than result disclosure alone, and we argue on that basis against minimally supported or direct-to-consumer provision of neurodevelopmental risk information.

## 12 Ethical implications

Ethical analysis must separate what is technically possible from what is clinically appropriate and ethically justified; the three do not coincide here.

Reproductive autonomy and its preconditions. Autonomous decision-making requires accurate information. Where predictive validity is low and uncertainty high, presenting genomic findings as a basis for reproductive decisions risks undermining the autonomy it purports to serve (48, 134, 135). Qualitative research on attitudes toward prenatal genetic testing for autism and decisions about affected pregnancies demonstrates wide divergence in views, including among people directly affected by autism (140), which argues for neutral provision rather than assumed preferences.

The ethics of complexity. A dedicated review argues that the probabilistic nature of autism genetics generates ethical problems that do not arise for highly penetrant Mendelian conditions (134). Applying frameworks developed for highly penetrant conditions to probabilistic neurodevelopmental risk may therefore be inappropriate without substantial modification.

Stigmatization, neurodiversity, and disability rights. Autism is both a source of significant support needs for some individuals and a form of human variation with which many autistic people identify; survey evidence indicates that self-identification as autistic and awareness of neurodiversity are associated with viewing autism as an identity rather than a condition requiring cure, while also showing substantial common ground with medical-model perspectives (141). The disability rights critique of prenatal testing holds that selecting against a trait can express a negative judgment about people living with that trait, and that assumptions about quality of life with disability are frequently mistaken (135). These considerations apply with particular force to a probabilistic neurodevelopmental finding. A balanced position acknowledges the legitimacy of both perspectives without adjudicating between them or presenting either as the default clinical stance.

Selective termination. Decisions to continue or end a pregnancy belong to the prospective parents. The obligation of the clinical and scientific community is to ensure such decisions are not made on the basis of overstated predictive claims. Where a finding is compatible with a wide range of outcomes including typical development (58, 59), presenting it as predictive of autism would materially distort decision-making.

Polygenic embryo selection and commercialization. Polygenic embryo screening is offered commercially despite modeling indicating limited expected utility (110) and evidence that physician decision criteria are heterogeneous (111). Ethical reviews document unresolved arguments about validity, equity, and consent (112, 113), and analyses of polygenic prediction for multifactorial disease highlight risks of misinterpretation (109). We therefore argue that commercialization of poorly validated prediction tools is a foreseeable extension of this pattern into the prenatal domain and warrants anticipatory regulatory attention.

Equity. Because genomic reference data and polygenic scores are skewed toward European-ancestry populations (99), any prenatal risk-estimation tool built on them would deliver unequal accuracy. Frameworks for responsible implementation of genomic screening emphasize duties of justice and adequate infrastructure alongside individual consent (142), implying that implementation should not precede demonstrated equitable performance.

## 13 Future directions

The priorities set out below are derived from the preceding synthesis; they are the assessment of this review rather than established recommendations of professional societies. Progress toward clinically useful prenatal risk characterization — not prenatal diagnosis — would require advances on several fronts. The gaps are specific and, taken together, define the research agenda: absent individual-level predictive validity for autism; incomplete variant-specific penetrance and expressivity estimates; unexplained phenotypic heterogeneity among carriers of the same variant; genomic discovery datasets that remain ancestrally narrow; few prospective prenatal-to-childhood longitudinal cohorts; little integration of rare variants, common polygenic background, and prenatal exposures within a single model; limited characterization of gene–environment interaction in humans; no clinically validated fetal molecular biomarker; no validated autism-specific prenatal prediction model; sparse evidence on real-world counseling outcomes; unresolved policy on variant-of-uncertain-significance interpretation and reporting; and limited long-term evidence on the psychosocial consequences of autism-related genetic testing. **Table 4** maps these gaps onto the stage of the four-stage framework at which each arises.

Larger and ancestrally diverse cohorts. Both rare-variant discovery (6, 7) and common-variant analysis (8) remain underpowered relative to autism’s heterogeneity, and diversification is a precondition for equitable prediction (99).

Less ascertainment-biased penetrance and expressivity estimates. The critical missing quantity is variant-specific, prospectively estimated penetrance in population-based rather than clinically ascertained samples. Current estimates derive largely from clinically ascertained cohorts and are consequently likely to be biased upward: carriers of the same recurrent CNVs have repeatedly been identified among unaffected transmitting parents and among individuals without apparent clinical phenotype, who would not enter a clinical series (33, 56, 58, 60). One important strategy for reducing this bias is population-scale follow-up of carriers identified outside clinically ascertained cohorts.

Integration of rare and common variation. Evidence that the two act additively (37) provides a rationale for testing whether combined models outperform either alone; such models would require joint estimation in the same cohorts and prospective validation.

Longitudinal prenatal-to-childhood cohorts. We suggest that designs linking prenatal genomic characterization, prenatal biological measures, and standardized developmental follow-up through the age of reliable diagnosis would provide the substrate for any validated prenatal risk model. This is a recommendation of the present review rather than a finding of the cited literature.

Multi-omic and single-cell developmental resources. Fetal transcriptomic atlases (69, 70) and single-cell developmental maps (71) should be extended and integrated with epigenomic and proteomic data to refine which developmental windows and cell types mediate risk.

Functional validation to close the interpretation gap. Systematic functional characterization — of the kind that clarified the directional effects of SCN2A alleles (46) and the regulatory role of CHD8 (44, 45) — is the most direct route from analytic detection to clinical interpretation. Cellular and organoid model systems (75) offer one route to scaling this work, though findings in such systems require confirmation in human tissue.

Gene–environment integration. Better characterization of interactions between genetic liability and prenatal biological environments (100, 101) is needed before such factors can contribute to risk models rather than only to uncertainty.

Methodological and governance standards. Ancestry-robust PRS methods and transparent reporting (103, 105, 108); consistent prenatal variant classification and agreed VUS reporting policy (49); prospective evaluation of whether returning neurodevelopmental risk information improves outcomes; and counseling and governance infrastructure developed alongside rather than after technical capability (16, 78, 142).

A more plausible research program than an “autism gene” or a prenatal autism test would be probabilistic, multimodal risk characterization — integrating rare variants, polygenic background, family history, and where validated, prenatal biological measures — that yields calibrated ranges rather than classifications, delivered within a counseling framework designed for uncertainty.

## 14 Conclusion

Autism genetics has produced substantial and durable knowledge: high-confidence risk genes from large-scale exome sequencing, recurrent CNVs with established associations, evidence that common variation contributes additively to liability, and convergent functional genomic evidence placing much of this biology within mid-fetal cortical development. Prenatal genomics has advanced in parallel, with CMA, ES, GS, and cfDNA-based approaches now able to characterize fetal genomes in unprecedented detail.

These advances make it possible to identify, before birth, specific genetic variants associated with elevated neurodevelopmental and autism susceptibility, and in some cases to establish a molecular diagnosis of a defined genetic condition. They do not make it possible to diagnose autism prenatally or to predict at the individual level whether a given fetus will later meet diagnostic criteria. What a fetal genomic finding permits clinicians and families to infer is therefore a range of outcomes, not an outcome. A pathogenic or likely pathogenic variant can establish a molecular diagnosis and, with it, the outcomes reported in carriers of that variant: a higher probability than in the general population of developmental delay, intellectual disability, epilepsy, autism, and other features, together with any implications for physical health, for parents, and for recurrence. For well-studied recurrent CNVs it can indicate approximate outcome frequencies — for example, about one in four predominantly clinically referred carriers of the 16p11.2 deletion met criteria for autism (Section 4.5) — but such figures derive largely from clinically ascertained samples and may overstate the probability for a fetus in whom the variant is found incidentally. It cannot specify which outcome, or what severity, will occur in a particular child; no calibrated individual probability of autism is yet available; a variant of uncertain significance supports no outcome inference; and a negative result does not exclude autism. Common polygenic variation currently shifts group-level odds modestly and does not support individual prenatal inference. Prenatal diagnostic genomic testing is, moreover, largely indication-driven and is neither designed nor validated as a population-level neurodevelopmental risk assessment.

The most scientifically defensible direction is therefore not a prenatal autism test but improved probabilistic risk characterization — better calibrated, ancestrally equitable, prospectively validated, and integrated with developmental and clinical follow-up — coupled with counseling standards and ethical safeguards that preserve the distinction between molecular diagnosis and autism diagnosis, and between susceptibility and outcome. Maintaining those distinctions is the condition under which prenatal genomic information can support families rather than sustain claims the evidence does not permit.
